# Fibroblast Growth Factor-10 (FGF-10) Mobilizes Lung-resident Mesenchymal Stem Cells and Protects Against Acute Lung Injury

**DOI:** 10.1038/srep21642

**Published:** 2016-02-12

**Authors:** Lin Tong, Jian Zhou, Linyi Rong, Eric J. Seeley, Jue Pan, Xiaodan Zhu, Jie Liu, Qin Wang, Xinjun Tang, Jieming Qu, Chunxue Bai, Yuanlin Song

**Affiliations:** 1 Department of Pulmonary Medicine, Zhongshan Hospital, Fudan University, Shanghai, 200032, P. R. China; 2 Division of Pulmonary and Critical Care Medicine, University of California, San Francisco, California 94143, USA; 3 Department of Pulmonary Medicine, Rui Jin Hospital, Shanghai Jiao Tong University School of Medicine, Shanghai, 200025, P. R. China; 4 State Key Laboratory of Respiratory Disease, Guangzhou Medical University, Guangzhou, 510120, P. R. China; 5 Shanghai Public Health Clinical Center, Shanghai, 201508, P. R. China; 6 Zhongshan Hospital, Qingpu branch, Fudan University, Shanghai, 201700, P. R. China

## Abstract

FGF-10 can prevent or reduce lung specific inflammation due to traumatic or infectious lung injury. However, the exact mechanisms are poorly characterized. Additionally, the effect of FGF-10 on lung-resident mesenchymal stem cells (LR-MSCs) has not been studied. To better characterize the effect of FGF-10 on LR-MSCs, FGF-10 was intratracheally delivered into the lungs of rats. Three days after instillation, bronchoalveolar lavage was performed and plastic-adherent cells were cultured, characterized and then delivered therapeutically to rats after LPS intratracheal instillation. Immunophenotyping analysis of FGF-10 mobilized and cultured cells revealed expression of the MSC markers CD29, CD73, CD90, and CD105, and the absence of the hematopoietic lineage markers CD34 and CD45. Multipotency of these cells was demonstrated by their capacity to differentiate into osteocytes, adipocytes, and chondrocytes. Delivery of LR-MSCs into the lungs after LPS injury reduced the inflammatory response as evidenced by decreased wet-to-dry ratio, reduced neutrophil and leukocyte recruitment and decreased inflammatory cytokines compared to control rats. Lastly, direct delivery of FGF-10 in the lungs of rats led to an increase of LR-MSCs in the treated lungs, suggesting that the protective effect of FGF-10 might be mediated, in part, by the mobilization of LR-MSCs in lungs.

Mesenchymal stem cells (MSCs) are adult connective tissue progenitor cells with multilineage differentiation potential^1^. In addition to bone marrow (BM), MSCs can also be isolated from adult, nonhematopoietic organs such as the lung^2^. These tissue-resident MSCs represent a reservoir of endogenous organ-specific adult progenitor cells with a potential role in local tissue homeostasis and repair. Potent immune-regulation and anti-inflammatory properties of MSCs have provoked significant interest in their application as vectors for tissue repair and cell therapy. Exogenously administered MSCs have been found to ameliorate injury in various animal models, including bleomycin-induced pulmonary fibrosis^3^, endotoxin-induced acute lung injury^4^, papain-induced emphysema^5^, and pulmonary hypertension^6^, as well as cardiac^7^ and renal allograft rejection^8^, and these reparative effects appear to be mediated by the secretory function of MSCs, rather their direct engraftment and growth. Lung-resident MSCs (LR-MSCs) can be isolated from bronchoalveolar lavage (BAL) of transplanted allografts, and were shown to be distinct from those derived from the bone marrow^9^. LR-MSCs have the ability to engraft in their organ of origin and to communicate with locally resident epithelial cells via gap junction communications^10^. Furthermore, LR-MSCs secrete KGF, an important epithelial growth factor, suggesting that lung derived MSCs can modulate alveolar epithelial cells in a paracrine fashion^10^. Allergen sensitization and challenge is accompanied by an increase of MSCs resident in the lungs that may regulate inflammatory and fibrotic responses as well^11^.

Fibroblast growth factor-10 (FGF-10), also known as keratinocyte growth factor-2 (KGF-2), has been shown to mediate epithelial-mesenchymal interactions, which are essential to lung development^12^,^13^,^14^,^15^,^16^,^17^,^18^. FGF-10 is a 20-kDa heparin-binding protein predominantly expressed by mesenchymal cells. It binds with high affinity to a spliced variant of fibroblast growth factor receptor 2-IIIb (FGFR2-IIIb), and also has a weaker affinity for FGFR1-IIIb^15^,^19^,^20^,^21^,^22^. Recently it was illustrated that FGF-10 could prevent lung injury after various stresses including bleomycin-induced pulmonary fibrosis^23^, high altitude pulmonary edema^24^, LPS-induced lung injury^25^, mechanical ventilation induced lung injury^26^ and ischemia-reperfusion lung injury^27^. Although the experimental data illustrating the protective effects of FGF-10 on lung injury are robust, the mechanism underlying these protective effects have not been fully elucidated.

In this study, we demonstrate that LR-MSCs can be easily isolated from the lower respiratory tract of rats pretreated with FGF-10. Additionally, we illustrate that the LR-MSCs isolated from FGF-10 pretreated rats are protective against LPS-induced acute lung injury. Collectively, we demonstrate for the first time the role of FGF-10 in LR-MSCs proliferation, mobilization and the organ specific protective effects against acute lung injury.

## Results

### Isolation of plastic-adherent fibroblastoid cells from BAL fluid of FGF-10 pretreated lungs

**Figure 1:**
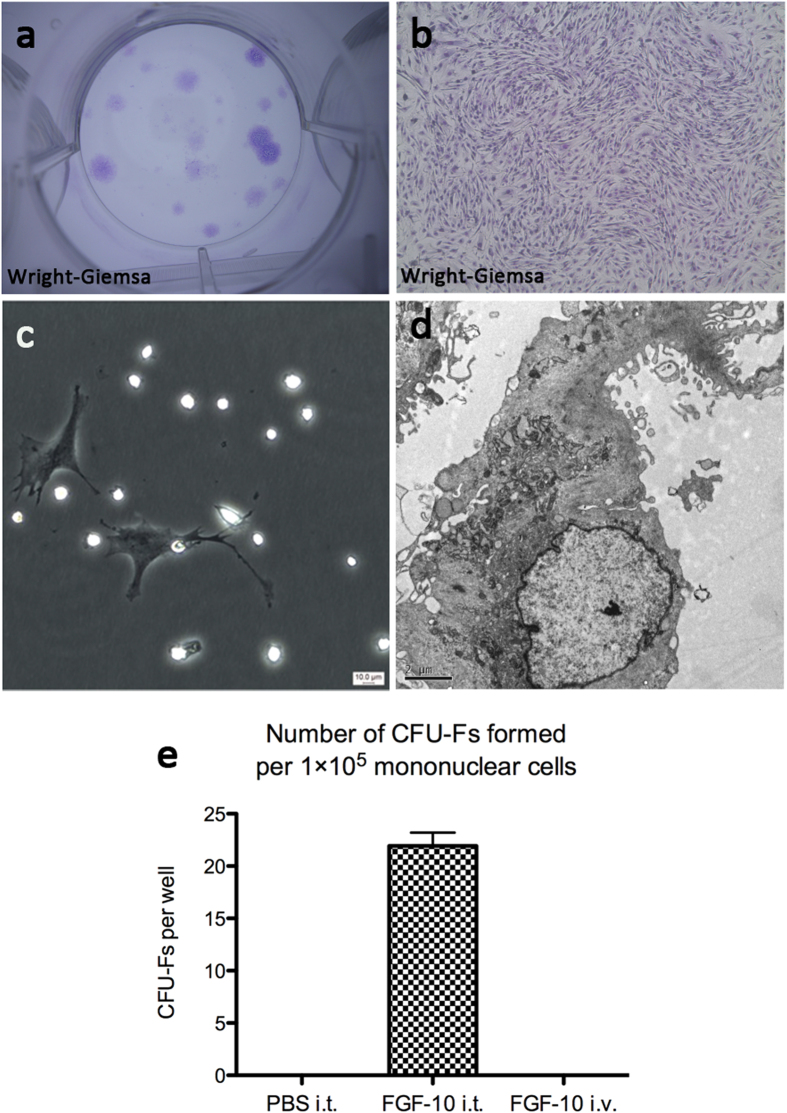
Isolation and characterization of fibroblastoid cells from BAL of rats pretreated with FGF-10. (**a**) Two weeks after plating of mononuclear cells obtained from BAL of FGF-10 pretreated rats, Wright-Giemsa staining identified the CFU-Fs. (**b**) An individual CFU-F with Wright-Giemsa staining. (**c**) Forty-eight hours after initial plating of mononuclear cells obtained from BAL of FGF-10 pretreated rats, fibroblastoid cells could be seen adherent to plastic bottom of culture flasks. (**d**) Electron microscopy imaging of the fibroblastoid cells from BAL. (**e**) Number of CFU-Fs formed per 1 × 10^5^ mononuclear cells collected via BAL from rats prereated with intratracheal PBS (PBS i.t.), intratracheal FGF-10 (FGF-10 i.t.), or intravenous FGF-10 (FGF-10 i.v.). Each point corresponds to the mean ± SEM.

Intratracheal instillation of FGF-10 (5 mg/kg) three days prior to inflammatory injury was demonstrated to be protective in models of acute lung injury^23^,^24^,^25^,^26^,^27^. To determine whether pretreatment with FGF-10 would mobilize MSC-like cells, FGF-10 was administered to rats and BAL fluid was collected and analyzed. Forty-eight hours after plating cells collected through BAL, adherent Fibroblastoid cells, which were much larger than alveolar macrophages, could be seen adhering to the bottom of plastic culture flasks (Fig. 1c,d). Fibroblastoid colonies were observed 14 days after cells collected via BAL were plated (Fig. 1a,b). The number of colony-forming unit-fibroblasts (CFU-Fs) formed per 1 × 10^5^ mononuclear cells plated varied between samples (mean, 22; range, 12–28) (Fig. 1e). CFU-Fs were identified in 100% (n = 15) of the BAL samples obtained from the lungs of rats pretreated with FGF-10. These CFU-Fs were expanded in culture by subsequent trypsinization and serial passage.

### Phenotypic characteristics of BAL-derived fibroblastoid cells

**Figure 2:**
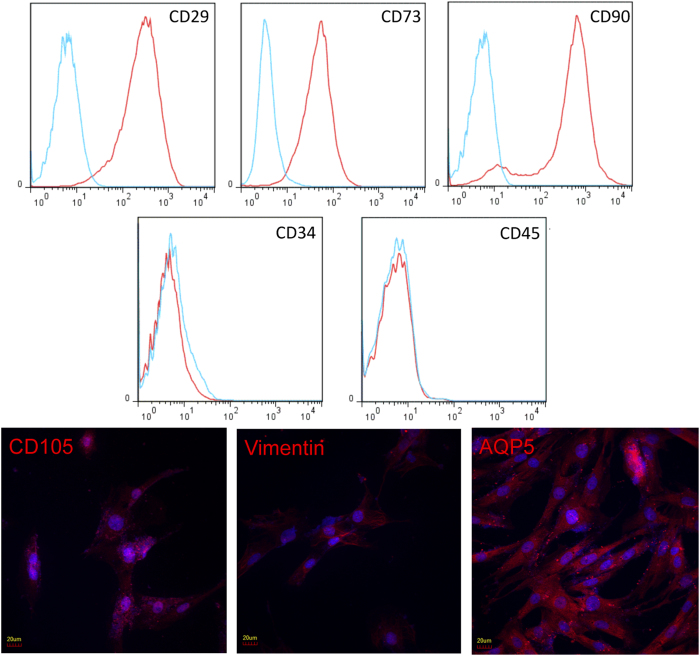
Immunophenotyping of BAL-derived fibroblastoid cells. Flow cytometric analysis was used to identify the surface antigens of BAL-derived fibroblastoid cells. These cells were predominantly positive for CD29, CD73 and CD90 (upper panels), and negative for the hematopoietic lineage markers CD34 and CD45 (middle panels). All histograms show specific mAbs in red and control isotype-specific IgGs in blue. Immunofluorescence staining of BAL-derived fibroblastoid cells demonstrated expression of CD105, AQP5 and vimentin (lower panels).

CFU-Fs initially identified from the BAL were expanded in cell culture without morphological evidence of cellular senescence. Immunophenotyping for surface antigens by flow cytometry was performed on BAL-derived CFU-Fs to further characterize these cells. They strongly expressed CD29, CD73 and CD90 (Fig. 2), as previously described for bone marrow-derived MSCs^28^. Furthermore, these cells did not express the hematopoietic lineage markers CD34 and CD45 (Fig. 2). Immunofluorescence studies of these BAL-derived cells demonstrated expression of the MSC marker CD105, the mesenchymal cell marker vimentin and the water channel aquaporin-5 (AQP5) as previously described for bone marrow-derived MSCs^29^ (Fig. 2).

### Multipotential differentiation of BAL-derived mesenchymal cells

**Figure 3:**
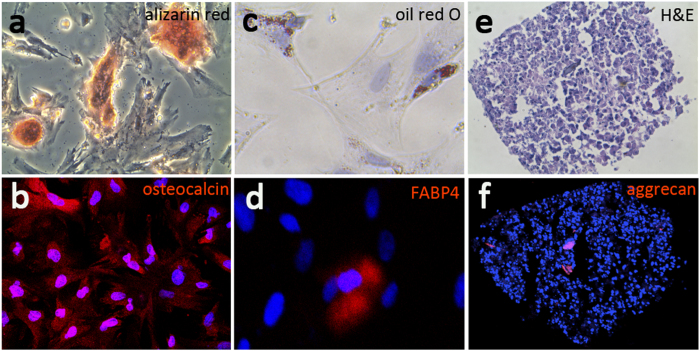
Mesenchymal cells isolated from BAL differentiate to multiple connective tissue lineages. (**a**) Osteocytic differentiation was indicated by calcium deposition as demonstrated by alizarin red staining. (**b**) Osteocytic differentiation was confirmed by inmunofluorescent staining of osteocalcin. (**c**) Adipocytic differentiation was demonstrated by accumulation of lipid droplets that stained positively with oil red O. (**d**) Adipocytic differentiation was confirmed by inmunofluorescent staining of FABP4. (**e**) Chondrocytic differentiated cells of pelleted micromass were stained with H&E. (**f**) Chondrocytic differentiation was evidenced by the presence of the extracellular matrix proteoglycan aggrecan.

One of defining characteristics of MSCs is their ability to differentiate into multiple mesenchymal lineages. Thus we determined the ability of BAL-derived mesenchymal cells to differentiate into specific connective tissue cell types. Mesenchymal cells (at passages 3–5) were studied under defined culture conditions to induce osteocytic, adipocytic, and chondrocytic differentiation (as described in Methods). The ability of BAL-derived mesenchymal cells to differentiate into osteocytes was demonstrated by staining for extracellular mineralization in cells following osteogenic stimulation (Fig. 3a), and was confirmed by inmunofluorescent staining of osteocalcin (Fig. 3b). Osteocytic differentiation was absent in control untreated cells. Adipocytic differentiation was demonstrated in cells stimulated with insulin and other adipogenic stimuli by accumulation of lipid-rich vacuoles that stained positively with oil red O (Fig. 3c), and was confirmed by immunofluorescent staining of FABP4 (Fig. 3d). No oil red O staining was seen in the control cells. Pelleted micromasses of cells treated with chondrocytic stimulation underwent chondrocytic differentiation, as evidenced by the presence of the extracellular matrix proteoglycan aggrecan (Fig. 3f). These studies suggest that BAL-derived mesenchymal cells are capable of differentiating into multiple connective tissue cell lineages.

### Microarray data of LR-MSCs

**Figure 4:**
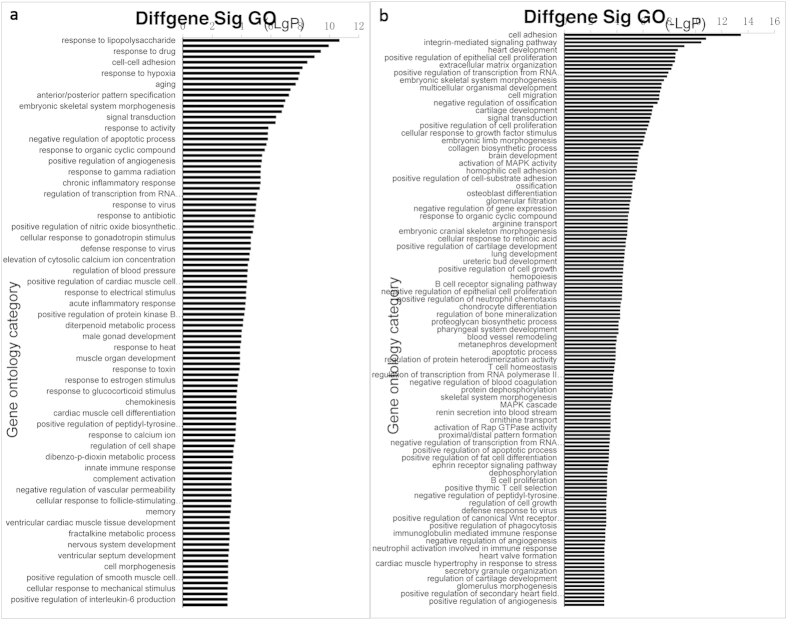
Gene Ontology (GO) analysis of the transcriptome revealed the functional diversity of BM-MSCs and BAL-derived MSCs (n = 3). (**a**) Compared with BM-MSCs, down-regulated functions of BAL-derived MSCs. (**b**) Compared with BM-MSCs, up-regulated functions of BAL-derived MSCs.

**Figure 5:**
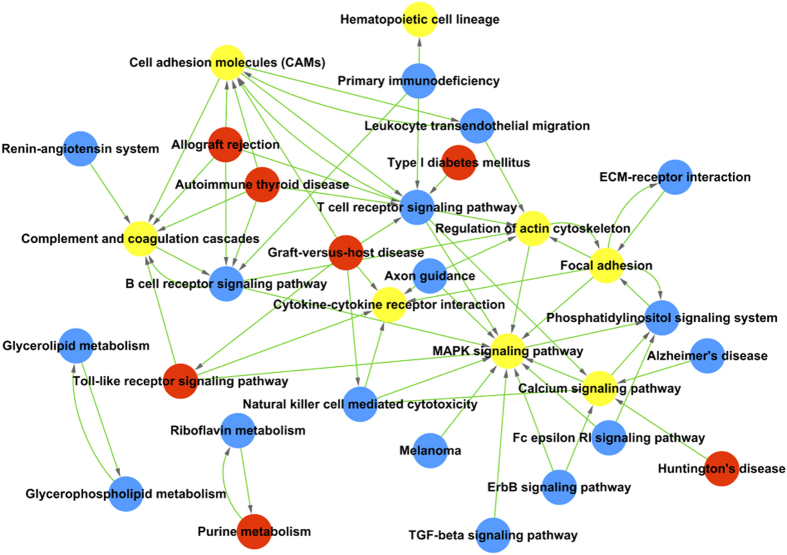
Pathway analysis of the transcriptome revealed the signaling pathway diversity of BAL-derived MSCs and BM-MSCs. Red circle represent the pathway of up-regulated genes, comparing to BM-MSCs. Blue circle represent the pathway of down-regulated genes, comparing to BM-MSCs. Yellow circle represent the pathway of both up- and down-regulated genes, comparing to BM-MSCs.

To determine whether the gene expression profiles in MSCs from the FGF-10 treated lungs are different from those derived from the bone marrow, we compared the transcriptome of MSCs from these two sources by Affymetrix analysis (GEO accession: GSE68243). Gene Ontology (GO) analysis showed that compared to BM-MSCs, BAL-derived MSCs had increased expression of genes involved in cell adhesion, integrin-mediated signaling pathways, epithelial cell proliferation, extracellular matrix organization, cell migration, *etc*, while other transcription programs were down regulated, including the response to lipopolysaccharide, response to drug, cell-cell adhesion, response to hypoxia, aging, *etc* (Fig. 4). Pathway analysis showed that MAPK signaling pathways were similarly regulated in both MSC sub-types (Fig. 5).

### EdU incorporation of LR-MSCs

**Figure 6:**
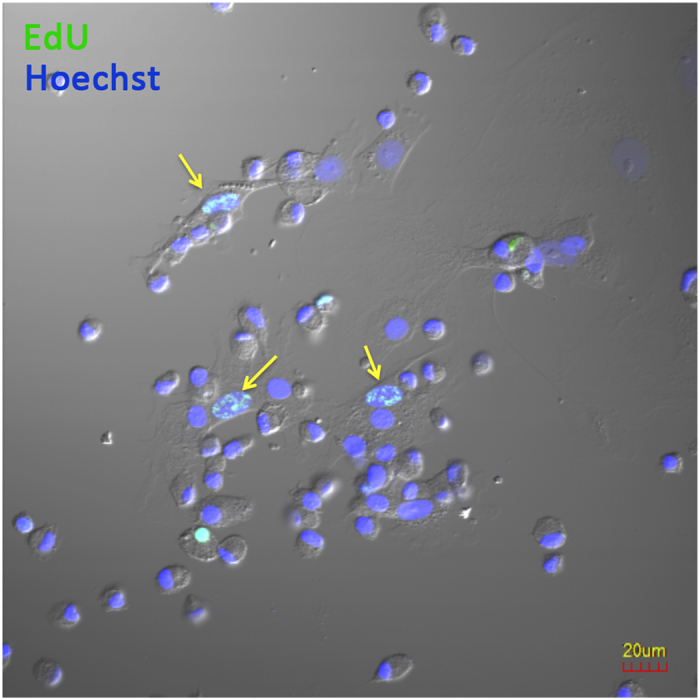
EdU incorporation assay. EdU was injected intraperitoneally to FGF-10 pretreated rats 24 hours before BAL. The mononuclear cells obtained from BAL of FGF-10 pretreated rats were cultured for 5–7 days, and detected for EdU incorporation. There were EdU positive MSCs among the cultured BAL-derived MSCs (arrow).

Lung tissue-specific MSCs are present in the adult lung^9^. To determine whether the increased number of MSCs in BAL of FGF-10 pretreated lungs represent the proliferation of MSCs, EdU (5-ethynyl-2′-deoxyuridine) was injected intraperitoneally in FGF-10 pretreated rats 24 hours before BAL. MSCs in BAL fluid were subsequently isolated, cultured for 5–7 days, then EdU incorporation was measured. There were about 27% EdU positive MSCs among the isolated MSCs (Fig. 6), suggesting these cells had active DNA synthesis during the 24-hour period prior to BAL.

With intravenous injection of FGF-10 at the same dose (5 mg/kg) or 2–3 folds higher, no LR-MSCs were found in BAL fluid (Fig. 1e). Furthermore, peripheral blood mononuclear cells from FGF-10 treated rats (including intratracheal instillation and intravenous injection) were collected and cultured. No LR-MSCs could be isolated from BAL with either route of injection. Taken together, these data suggest that FGF-10 stimulates proliferation of lung resident MSCs, rather than mobilizing MSCs from bone marrow.

### Expression of FGF-10 receptors on MSCs

**Figure 7:**
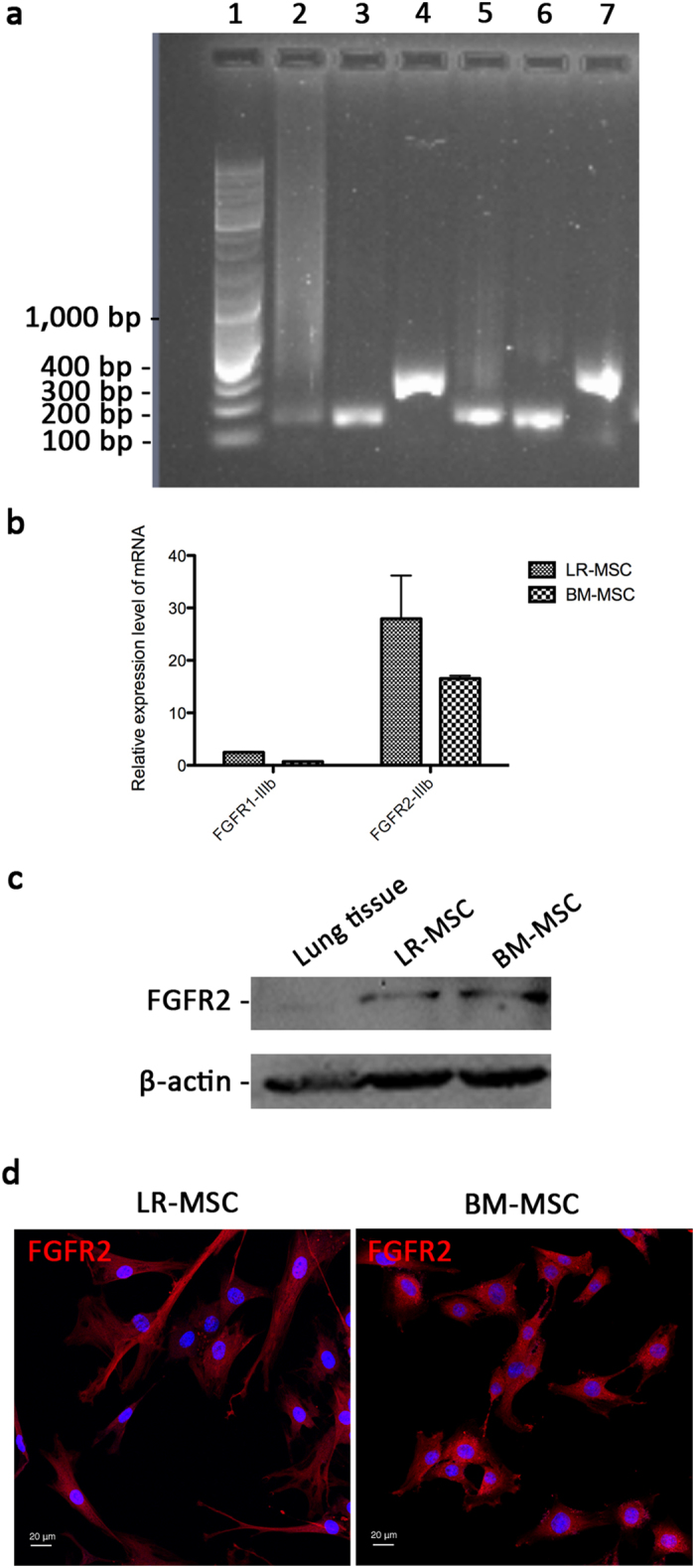
Expression of FGFR1-IIIb and FGFR2-IIIb in MSCs. (**a**) Real-time PCR was done with primers specific to FGFR1-IIIb and FGFR2-IIIb, and the PCR products were identified by electrophoresis (1, DNA marker; 2, β-actin of LR-MSCs; 3, FGFR1-IIIb of LR-MSCs; 4, FGFR2-IIIb of LR-MSCs; 5, β-actin of BM-MSCs; 6, FGFR1-IIIb of BM-MSCs; 7, FGFR2-IIIb of BM-MSCs). (**b**) The relative expression level of FGFR1-IIIb and FGFR2-IIIb in LR-MSCs and BM-MSCs. (**c**) Western blot analysis confirmed that both LR-MSCs and BM-MSCs express the protein of FGFR2. (**d**) Immunofluorescence staining of LR-MSCs and BM-MSCs demonstrated expression of FGFR2 on cell membrane. Each point corresponds to the mean ± SEM.

FGFR1-IIIb and FGFR2-IIIb are two receptors of FGF-10. To determine whether MSCs express these receptors, real-time PCR was done with primers specific to FGFR1-IIIb and FGFR2-IIIb, and the PCR products were sequenced to validate the specificity. It was shown that MSCs express the mRNAs of both receptors (Fig. 7a). Relative quantification data illustrated that FGFR2-IIIb expression is more abundant than FGFR1-IIIb at transcriptional level (Fig. 7b). Western blot analysis and immunofluorescence studies then confirmed that MSCs express the protein of FGFR2 on cell membrane (Fig. 7c,d).

### Effects of LR-MSCs and BM-MSCs on LPS-induced acute lung injury

BM-MSCs were found to be protective in several models of lung injury, including LPS-induced acute lung injury^30^. To determine whether LR-MSCs derived from BAL are also protective, LR-MSCs were delivered intratracheally to the lungs 4 hours after LPS instillation, and measures of lung injury were assessed 24 h and 48 h after LPS challenge.

**Figure 8:**
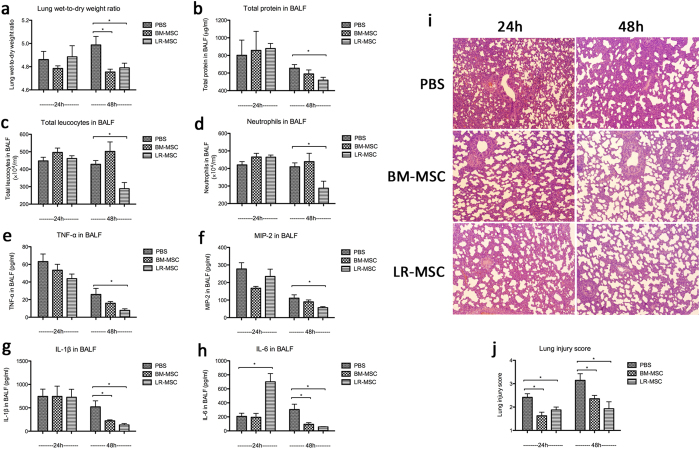
Effects of LR-MSCs and BM-MSCs on LPS-induced acute lung injury. LR-MSCs (BM-MSCs as positive control, and PBS as negative control) were intrapulmonary delivered to lungs 4 hours after LPS instillation, and the lung injury indices were examined 24 h and 48 h after LPS challenge. (**a**) Rats given MSCs showed a trend toward lower lung wet-to-dry weight ratio at 24 h, and a significant difference at 48 h. (**b**) BAL protein was not significantly reduced in the MSCs groups at 24 h, but significantly reduced in the LR-MSCs group at 48 h. (**c**,**d**) Total cell counts and absolute neutrophil counts were not different among the three groups at 24 h, but were significantly reduced in the LR-MSCs group at 48 h. (**e–h**) At 24 h, the levels of TNF-α, MIP-2, IL-1β and IL-6 in the BAL were not significantly lower in the MSCs-treated rats compared with the PBS-treated rats, and contrarily IL-6 showed a 3-fold increase in the BAL of the LR-MSCs treated rats. At 48 h, TNF-α and MIP-2 levels showed a trend toward being lower in the BAL of BM-MSCs treated rats, and were significantly lower in the BAL of the LR-MSCs treated rats. IL-1β and IL-6 levels in the BAL were significantly lower in both the BM-MSCs and LR-MSCs groups compared with the PBS group. (**i,j**) At both 24 h and 48 h, H&E staining of lung sections from MSCs-treated rats had significantly less injury compared with rats given PBS. Each point corresponds to the mean ± SEM. *P < 0.05.

Rats receiving BM-MSCs showed a trend toward lower lung wet-to-dry ratio at 24 h. And both the BM-MSCs and LR-MSCs groups showed a significant reduction in wet-to-dry ratio at 48 h compared to PBS control group (Fig. 8a). In addition, BAL protein, a marker of endothelial and epithelial permeability, was not significantly reduced in either MSCs groups at 24 h, but significantly reduced in the LR-MSCs group at 48 h compared to PBS control group (Fig. 8b).

BAL studies were done at 24 and 48 h after LPS instillation to determine whether neutrophil counts were lower in the MSCs-treated vs. PBS-treated rats. Total cell counts and absolute neutrophil counts were not different among the three groups at 24 h, but were significantly decreased in the LR-MSCs group at 48 h (Fig. 8c,d). At both 24 h and 48 h, H&E staining of lung sections from MSCs-treated rats had significantly less injury compared with rats given PBS (Fig. 8I,j).

At 24 h, the levels of TNF-α, MIP-2, IL-1β and IL-6 in the BAL were not significantly lower in the MSCs-treated rats compared with the PBS-treated rats. Surprisingly, IL-6 showed a 3-fold increase in the BAL of the LR-MSCs treated rats. At 48 h, TNF-α and MIP-2 levels showed a trend toward being lower in the BAL of BM-MSCs treated rats, and were significantly lower in the BAL of the LR-MSCs treated rats. IL-1β and IL-6 levels in the BAL were significantly lower in both the BM-MSCs and LR-MSCs groups compared with the PBS group (Fig. 8e–h).

## Discussion

In this study, we demonstrate that fibroblast-like cells can be isolated with a high yield from the distal airways of rats treated with FGF-10. BAL-derived mesenchymal cells exhibited several characteristics similar to classical bone marrow-derived MSCs^28^. First, they can be isolated by adherence purification on tissue culture plastic and form distinct CFU-Fs in cell culture. Second, these cells express surface markers typically associated with MSCs, but do not express hematopoietic stem cell markers. Third, these cells are multipotent and give rise to adipocytes, chondrocytes, and osteocytes. Additionally, the LR-MSCs isolated from FGF-10 pretreated lungs were protective against LPS-induced acute lung injury.

Previous studies of sex-mismatched human allografts have demonstrated that MSCs in lungs originate in the engrafted organ rather than in the bone marrow^9^. Furthermore, lung-resident MSCs differ from BM-derived MSCs with respect to their cytokine/chemokine gene expression profiles, confirming that these cells are distinct from those derived from the bone marrow^9^. Our data provide further evidence that LR-MSCs are located in lungs, and when stimulated with FGF-10 *in vivo*, these cells proliferate. Transcriptional profiles of LR-MSCs and BM-MSCs suggest important differences between these cells in terms of cell function and signaling pathways. These resident MSCs may thus represent a reservoir of endogenous organ-specific adult progenitor cells with a potential role in local tissue homeostasis and repair. When lung was injured, those organ specific progenitor cells proliferate and spread to injured area to repair.

Several studies illustrate that FGF-10 pretreatment protects against lung injury from various stresses, including bleomycin^23^, high altitude pulmonary edema^24^, ischemia reperfusion^27^, mechanical ventilation^26^, and LPS^25^. In this study, we illustrate that intratracheal administration of FGF-10 mobilized LR-MSCs, which could easily be collected by bronchoalveolar lavage. After collection, these LR-MSCs were cultured *in vitro* and then intratracheally delivered as cellular therapy to rats after LPS-induced lung injury with substantial beneficial effects. BM-MSCs were used as positive control, and PBS as negative control. We showed that at 48 hours, both LR-MSCs and BM-MSCs improved LPS-induced lung injury, and LR-MSCs seemed to be more effective than BM-MSCs in this scenario.

Although type II cell proliferation, increased surfactant production, reduced endothelial and epithelial apoptosis and enhanced macrophage endocytosis have been proposed to explain the therapeutic effects of FGF-10 in various animal models; however, the impact of FGF-10 on LR-MSCs could also be a potential mechanism for the protective effects of FGF-10 on lung injury. The kinetics of both LR-MSCs mobilization after FGF-10 delivery and the peak of the protective effects of direct delivery of FGF-10 to lung injured rats are temporally correlated, further suggesting that LR-MSCs mobilization may be one of the key mechanisms through which FGF-10 is lung protective.

Numerous studies have shown that MSCs can be protective against lung injury and can aid in lung repair^30^,^31^. Bone marrow-derived MSCs have been utilized more frequently in animal models illustrating the protective effects of MSC. In addition, a phase II trial of MSCs in human acute lung injury is currently underway. However, more than 99% of the MSCs injected into the lungs could not engraft, Thus, many studies suggest that paracrine mediators (including KGF, PGE2, IL1RA, *etc*.) secreted by MSCs are responsible for the protective effects during acute lung injury^31^. In contrast to bone marrow-derived MSCs, human lung-derived MSCs have the ability to engraft in the lung and to communicate with resident epithelial cells via gap junction communications^10^. Human LR-MSCs demonstrated long-term persistence in the murine lungs in which they were injected and remained engrafted in lung tissue up to 6 months after administration^10^. Both BM-MSCs and LR-MSCs secrete KGF, an important epithelial growth factor, suggesting that MSCs, regardless of tissue of origin, can modulate alveolar epithelial cells in a paracrine fashion^10^,^30^,^31^. Our data also demonstrate that BM-MSCs and LR-MSCs are protective against LPS induced lung injury when delivered after LPS administration. In addition, it appears that LR-MSCs may more effectively treat LPS mediated lung injury, which may be due to their superior ability to engraft.

It was shown that MSCs express FGFR2-IIIb, the receptor of FGF-10, and FGF-10 may activate MSCs by binding to FGFR2-IIIb. However, *in vitro* study revealed that MSCs did not proliferate more actively when stimulated by FGF-10 (data not shown). It seems that MSCs need the *in vivo* context to respond to FGF-10 stimulation. Further study may focus on the FGF-10 mediated intercellular communication and activation, which might fully activate the MSCs located in lungs. In addition, the exact location of LR-MSCs needs to be identified, whether it is transformed from Clara cell, type II cell, basal cell or fibroblast like cells from the airways needs further exploration.

In summary, this study demonstrates, for the first time to our knowledge, the isolation and characterization of LR-MSCs from the lower respiratory tract of adult healthy rats by pretreatment with FGF-10. Identification and characterization of these MSCs could represent the first step to understanding the mechanisms of FGF-10 in lung injury protection and repair and it also provide a new strategy to collect autologous MSCs for future clinical investigation.

## Methods

### Animals

Male Sprague-Dawley rats, 6–8 weeks old, bred under pathogen free conditions were used in these experiments. Animals were maintained in the animal facility at Fudan University with clean, controlled temperature and independent ventilation environment. The animals had free access to food and water.

### Animal treatment

Rats were anesthetized with intraperitoneal injection of chloral hydrate (300 mg/kg), and then fixed at a 60° angle on a table in a supine position. A fiber optic light source was placed immediately over the neck, and the oropharynx was lifted with forceps, allowing for the direct visualization of the trachea. The instillate (FGF-10, PBS, MSCs, or endotoxin) was then injected into the trachea using an 18G catheter attached to a 1-ml syringe as preciously described^32^. FGF-10 (provided by Newsummit Co., Shanghai, China) at a dose of 5 mg/kg was instilled through the catheter into the lungs of rats. Control animals received equal volume of PBS. Three days after FGF-10 or PBS instillation, the rats were sacrificed with an intraperitoneal injection of urethane (1.5 g/kg) and exsanguinated through the femoral artery. BAL was then performed immediately through tracheal intubation and repeated 5 times, and each time 5 ml medium (4 °C) was slowly infused, after which the fluid was slowly withdrawn and reinfused for additional two times. The medium used for BAL consists of DMEM/F12 supplemented with 5% fetal bovine serum (Invitrogen), 100 U/ml penicillin/streptomycin (Invitrogen), and 2.5 ug/ml amphotericin B (Invitrogen), 0.02 wt% EDTA (Invitrogen).

For LPS-induced ALI model, rats were intratracheally injected with either 5 mg/kg LPS (Escherichia coli O55:B5; Sigma, St. Louis, MO) dissolved in 0.3 ml PBS or vehicle (PBS). After 4 hours, 2 × 10^6^ MSCs (BAL-derived or BM-derived (Cyagen Biosciences, CA, USA)) dissolved in 0.3 ml PBS or vehicle (PBS) were instilled through the catheter into the lungs of rats. The rats were sacrificed 24 or 48 hours after LPS instillation with an intraperitoneal injection of urethane (1.5 g/kg).

### Isolation and culture of cells from BAL fluid

Recovered BAL fluid was centrifuged at 800 rpm for 10 minutes at 4 °C, and the pellets were resuspended and seeded at a density of 1 × 10^5^ mononuclear cells per well in 6-well cell culture plate. The cells were maintained in medium consisting of DMEM/F12 supplemented with 10% fetal bovine serum (Invitrogen), 2 mmol/l L-glutamine (Invitrogen), 100 U/ml penicillin/streptomycin (Invitrogen), and 2.5 ug/ml amphotericin B (Invitrogen) and incubated at 37 °C in 5% CO_2_/95% air. Medium was changed first after 24 hours and then every 3 days. Single separated fibroblastoid colonies termed CFU-Fs were identified 14 days after initial plating. For staining and enumeration of CFU-Fs, adherent CFU-Fs colonies were fixed with 10% formaldehyde and stained with Wright-Giemsa stain (Jiancheng, Nanjing, China). To study mesenchymal cells obtained from an individual rat, all colonies growing in 6-well cell culture plate were trypsinized and passed into a 100-mm plate. A homogeneous population of mesenchymal cells was obtained from individual rats after 3–5 passages.

### Assays for adipogenic, chondrogenic, and osteogenic differentiation

Multilineage differentiation to adipocyte, chondrocyte, and osteocyte lineages was tested in BAL-derived CFU-Fs at passage 5 using the rat mesenchymal stem cell functional identification kit (R&D systems, Minneapolis, MN, USA) according to the manufacturer’s instructions. Briefly, for adipogenic differentiation, confluent cell cultures in a 24-well plate were treated with adipogenic differentiation medium. At 3 weeks cells were fixed with 10% formaldehyde and incubated with fresh oil red O, lipid droplets were visualized and photographed. Immunofluorescence staining for FABP4 using anti-rat FABP4 antibody was performed to demonstrate adipogenic differentiation. Osteogenic differentiation was induced by incubating the cells with osteogenic differentiation medium. After 21 days, cells were fixed and incubated with freshly made 2% alizarin red stain. Immunofluorescence staining for osteocalcin using anti-rat osteocalcin antibody was performed to demonstrate osteogenic differentiation. For chondrogenic differentiation, cells were cultured as a pelleted micromass in chondrogenic differentiation medium. After 4 weeks, pellets were embedded en bloc in OCT, and 6-μm frozen sections were obtained. The sections were stained with H&E, and immunofluorescence staining for the extracellular matrix proteoglycan aggrecan using anti-rat aggrecan antibody was performed to demonstrate chondrocyte differentiation.

### Multiparameter flow cytometric analyses (FACS)

Cell-surface antigen phenotyping was performed on cell lines generated from individual rats at passage 5 using antibodies against CD34 (Santa Cruz, sc-7324), CD45 (eBioscience, 17-0461-80), CD29 (BioLegend, 102206), CD73 (BD, 551123), and CD90 (eBioscience, 12-0900-81). Briefly, cells were trypsinized and aliquoted at a concentration of 0.5 × 10^6^ cells/ml and stained for 30 minutes with either conjugated specific antibodies or isotype-matched control IgGs at recommended concentrations. Labeled cells were washed twice, resuspended in FACS buffer, and analyzed on flow cytometer (BD).

### Immunofluorescence staining

Immunofluorescence labeling was performed at passage 5 using mouse mAb against CD105 (Santa Cruz, H-300), vimentin (Cell Signaling Technology, R28), AQP5 (Abcam, ab78486), and FGFR2 (Abcam, ab10648). Cells were visualized and photographed using an Olympus confocal fluorescence microscope.

### RNA isolation and analysis

Total RNA was isolated from mesenchymal stem cells using the RNeasy Mini Kit (QIAGEN) as per the manufacturer’s instructions. Real-time PCR was performed for expression of FGFR1-IIIb and FGFR2-IIIb using the primers as previously described^33^. Primers for FGFR1-IIIb: forward 5′-CGGGGATTAATAGCTCGGATG-3′, reverse 5′-GCACAGGTCTGGTGACAGTGA-3′. Primers for FGFR2-IIIb: forward 5′-GAGCACCGTACTGGACCAACAC-3′, reverse 5′-TGGTAGGTGTGGTTGATGGACC-3′. β-Actin was used as a control, and the primers were forward 5′-GGAGATTACTGCCCTGGCTCCTA-3′, and reverse 5′-GACTCATCGTACTCCTGCTTGCTG-3′. The PCR reactions were carried out for 45 cycles with the primer sequences. The reaction products were resolved by electrophoresis on a 1.0% agarose gel and visualized with ethidium bromide. Further sequencing was performed to validate the PCR products.

For Affymetrix analysis, BM-MSCs were isolated from the normal rat bone marrow as previously described^34^. Total RNA was prepared from 3 sets each of bone marrow- and BAL-derived MSCs cultured under similar conditions at passage 5. Affymetrix array hybridization and scanning were performed by the Affymetrix and cDNA Microarray Core Facility at the Qiming biotech co., using Rat Gene 1.0 ST chips. Expression values for each gene were calculated using a robust multiarray average algorithm and expressed as log2-transformed data. Gene Ontology analysis was used to determine differential functions and signaling pathways between lung-derived and bone marrow-derived MSCs.

### EdU incorporation assay

EdU (5-ethynyl-2′-deoxyuridine) (Invitrogen) was injected intraperitoneally (100 mg/kg) to FGF-10 pretreated rats 24 hours before BAL, and the MSCs in BAL fluid were isolated and cultured for 5–7 days, then were detected for EdU incorporation according to manufacturer’s recommendations.

### Western blot analysis

Cells and homogenized lung tissue were lysed by RIPA and 20 μg of protein were electrophoresed. Membranes were exposed overnight at 4 °C to rabbit anti-FGFR2 polyclonal antibody (Abcam, ab10648) diluted 1:2000 or anti-β-actin antibody (Abcam, ab8227) diluted 1:1000 as loading control, then incubated for 1 h with horseradish peroxidase-conjugated goat anti-rabbit IgG antibody (Jackson ImmunoResearch, USA) diluted 1:20000.

### Wet-to-dry weight ratio

The right main bronchus was ligated, and the right lungs were excised. After wet weights were measured, the middle lobe of the right lung were placed in an oven at 60 °C for 72 h to allow determination of the wet-to-dry weight ratio.

### Bronchoalveolar Lavage

The bronchoalveolar lavage (BAL) was performed in the left lung. 2 ml PBS (4 °C) was slowly infused, after which the fluid was slowly withdrawn and reinfused for additional two times. The recovered fluid was collected for further analysis.

### Total cell count and differential cell count

BAL fluid (BALF) samples were centrifuged at 1200 rpm for 10 min at 4 °C, the supernatant was removed and stored at −80 °C. The pellet was resuspended in 0.5 ml of PBS. The total number of nucleated cells in BAL fluid was counted with a hemocytometer. Then the resuspended BAL fluid was centrifuged onto slides (1800 rpm for 15 min) and stained with Wright-Giemsa stain (Jiancheng, Nanjing, China). The slides were quantified for neutrophils number by counting a total of 200 cells per slide.

### BALF protein concentration

BALF protein concentration was measured using a bicinchoninic acid (BCA) protein assay kit according to the manufacturer’s instruction (Thermo Fisher Scientific, MA, USA).

### Cytokines in BALF

TNF-α, IL-1β, IL-6, and MIP-2 levels in BALF were measured using rat TNF-α, IL-1β, IL-6, and MIP-2 ELISA kits (R&D Systems, Minneapolis, MN) according to the manufacturer’s recommendations, respectively.

### Lung morphometry analyses

Histopathologic changes induced by LPS were evaluated in rats pretreated with MSCs or vehicle. The lungs were fixed with 10% formalin, and 5 μm sections were cut for H&E staining. The injury degree was scored with the following criteria: 1, no injury; 2, injury to 25% of the field; 3, injury to 50% of the field; 4, injury to 75% of the field; 5, diffuse injury^25^. All samples were examined by 3 pathologists blinded to the experimental procedures and the mean score was used.

### Statistics

Each point corresponds to the mean ± SEM. Statistical differences were determined using the one-way analysis of variance (ANOVA), and *p* < 0.05 was considered significant. Individual groups were compared using the unpaired Student’s *t* test.

### Study approval

All experimental protocols were approved by the Institutional Animal Care and Use Committee of Zhongshan Hospital, Fudan University. All animals were handled in accordance with the Guide for the Care and Use of Laboratory Animals published by the National Institutes of Health, and efforts were made to minimize suffering and pain of the animals, and number in each group in our study.

## Additional Information

**How to cite this article**: Tong, L. *et al.* Fibroblast Growth Factor-10 (FGF-10) Mobilizes Lung-resident Mesenchymal Stem Cells and Protects Against Acute Lung Injury. *Sci. Rep.* **6**, 21642; doi: 10.1038/srep21642 (2016).
